# A state-space modelling approach to wildlife monitoring with application to flying-fox abundance

**DOI:** 10.1038/s41598-018-22294-w

**Published:** 2018-03-06

**Authors:** David A. Westcott, Peter Caley, Daniel K. Heersink, Adam McKeown

**Affiliations:** 1 CSIRO Land and Water, PO Box 780, Atherton, Queensland Australia; 2 CSIRO Data61, GPO Box 1700, Canberra, ACT 2601 Australia; 3 CSIRO Land and Water, PO Box 12139, Earlville BC, Qld 4870 Australia

## Abstract

Monitoring flying-foxes is challenging as their extreme mobility produces highly dynamic population processes, considerable logistic difficulty, and variability in estimated population size. We report on methods for inferring population trend for the population of the spectacled flying-fox (*Pteropus conspicillatus*) in Australia. Monthly monitoring is conducted at all known roost sites across the species’ range in the Wet Tropics Region. The proportion of animals in camps varies seasonally and stochastic environmental events appear to be influential. We develop a state-space model that incorporates these processes and enables inference on total population trends and uses early warning analysis to identify the causes of population dynamics. The model suggests that population growth rate is stable in the absence of cyclones, however, cyclones appear to impact on both survival and reproduction. The population recovered after two cyclones but declined after a third. The modelling estimates a population decline over 15 years of *c*. 75% (mean *r* = − 0.12*yr*^−1^ and belief of negative trend is *c*. 83%) suggesting that conservation action is warranted. Our work shows that a state-space modelling approach is a significant improvement on inference from raw counts from surveys and demonstrates that this approach is a workable alternative to other methods.

## Introduction

Monitoring is critical to threatened species management as it provides baseline data about a species’ abundance, distribution, and, over time, trends in these^1,2^. As a consequence monitoring data are central to the development of management plans and, in the case of contentious species, to associated public debate. Since monitoring is so important, a great deal of attention has focused on developing sampling designs and analytical methods that improve its accuracy and reliability^3–6^. Monitoring results are essentially inferences about the outcomes of processes operating in two realms; the focal species’ population dynamics, and, the process of observing and recording those dynamics. The nature of these processes can dramatically influence the reliability of estimates and the reliability of inferences drawn from them. As a consequence, separating population signal from biological (process) noise and observation error is critical. It is also a fundamental challenge. State-space models (SSMs) are quantitative models that are increasingly used to infer population dynamics from time-series data, with the attraction of facilitating the coupling of a mechanistic model including process noise with an observation model that includes uncertainty^7^. A primary advantage of a SSMs, and in particular the Bayesian implementation we present here, is that they allow the incorporation of reasonably complex population and observation processes (including current levels of understanding of these), along with stochastic high impact events such as tropical cyclones, into an inferential model based on a single time series. That said, the ability to estimate parameters is constrained by model identifiability, which is typically influenced by the length of the time series, the extent of process driven variation within the data, and the inherent information content in relation to the parameters being estimated^8^.

There is an increasing need to monitor flying-fox (Chiroptera: Pteropodidae) populations. Right across their range, flying-foxes species are a focus of management^9–12^ due to their impacts on crops^13^, on amenity^14,15^, and concerns about their role as vectors of disease^16,17^. Persecution and habitat loss have seen global declines in flying-fox populations^9–11^ and currently 60% of Pteropus spp. for which data are available are listed as Vulnerable, Endangered or Critically Endangered in The IUCN Red List^18^. Unfortunately, monitoring of flying-foxes is not logistically straightforward, and as a consequence there are only a few species for which formal monitoring programs have been established. The extreme mobility of both individuals^19^ and populations^20,21^ means that monitoring must be done at the scale of the entire population or the species’ range^22^. In Australia’s National Flying-Fox Monitoring Program^21,23^, monitoring is conducted at the scale of the eastern seaboard of the continent and monitoring is conducted at roosts (hereafter called camps).

Flying-fox monitoring is subject to observational and process uncertainties that arise due to ecological and observational processes. Flying-foxes are not always found in known camps. During the summer months the majority of flying-foxes overnight in camps (e.g. telemetry studies^21^ suggest c. 80% for *Pteropus conspicillatus*), but during the cooler months a large and variable proportion of the population leave the camps to roosts at unknown locations (c. 90%^21^). Camps are also sometimes missed during a census due to logistical constraints, e.g. availability of counters, or the loss of access. Additionally, severe weather events, including tropical cyclones and prolonged heat waves, occasionally occur in the range of flying-foxes, potentially disrupting movement and roosting patterns and killing large numbers^20,24^. A method to satisfactorily estimate the abundance and trends of flying-foxes must attempt to accommodate all of these complex issues.

The spectacled flying-fox (*Pteropus conspicillatus*) provides an ideal system to test sampling designs and associated models for estimating population abundance for species in the family Pteropodidae. In Australia the species is almost entirely geographically restricted to the Wet Tropics of Northern Queensland, Australia, and there is little interchange with New Guinea or Moluccan populations^25^. A population is known to occur on Cape York but this is small (a few hundred individuals) and ephemeral (being present in certain months and some years)^26^. Thus the Wet Tropics population represents the vast bulk of the species’ Australian population and has been subject to intensive survey effort since 2004^20,27^. Within its distribution it exhibits qualitatively similar movement patterns to other flying-fox species with much wider distributions, namely constantly changing patterns of distribution across camps over time.

In this paper, using the Australian population of the spectacled flying-fox as our focal species, we use a SSM as the basis for estimating trends in the abundance of this highly mobile species in which the proportion of the population available to be counted varies over time. This approach maximises the information content from the available monitoring data which spans the period 2004–2017. To do this we use data on spectacled flying-foxes to estimate the effect of different proportions of flying-fox individuals being resident in unsurveyed or unknown camps on estimates of abundance based on surveys of known camps. In doing so, we develop and test such modelling methods for inferring trends in the abundance of flying-foxes in other monitoring programs, e.g. grey-headed flying-fox (*Pteropus poliocephalus*) in Australia’s National Flying-Fox Monitoring Program^21^, and elsewhere.

## Results

### Population monitoring

**Figure 1:**
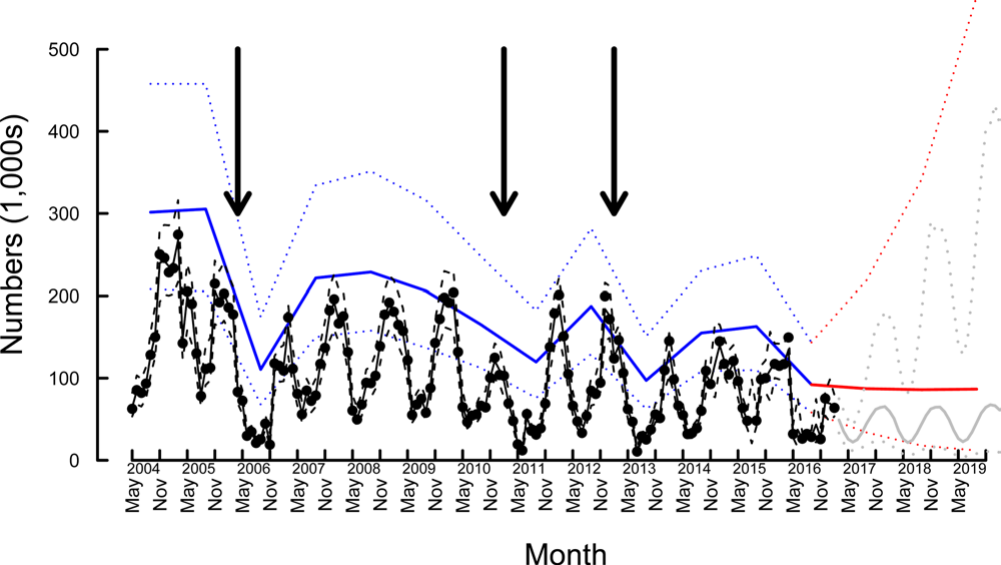
95% posterior predictive intervals for November total of spectacled flying-fox population (blue) and spectacled flying-fox population found in camps (black) utilizing all known camps (*n* = 62). The solid lines indicate the medians, and dotted lines the 95% credibility intervals. The black dots are observed counts of spectacled flying-foxes in camps, with dashed lines the 95% credibility intervals. The timing of major cyclones are indicated by the black arrows (from left to right, Cyclones Larry, Yasi, and Oswald). Projections (in the absence of cyclones) are shown in red for the total population and grey for the in-camp population.

Over the period 2004–2017 up to 154 monthly counts were conducted at each of 64 camps distributed across the Wet Tropics Region. In any given month an average of 10.5 ± 2.4 (± S.D.) camps were occupied. The total estimated *P*. *conspicillatus* population shows very strong annual dynamics with low numbers of animals counted in camps during the cooler months (April to September) and population peaks during the warmer months (November to February) (Fig. 1). Over the course of the monitoring the counted November population decreased from 250,270 in 2004 to 75,347 in 2016 (Fig. 1).

#### Drivers of perturbations

**Figure 2:**
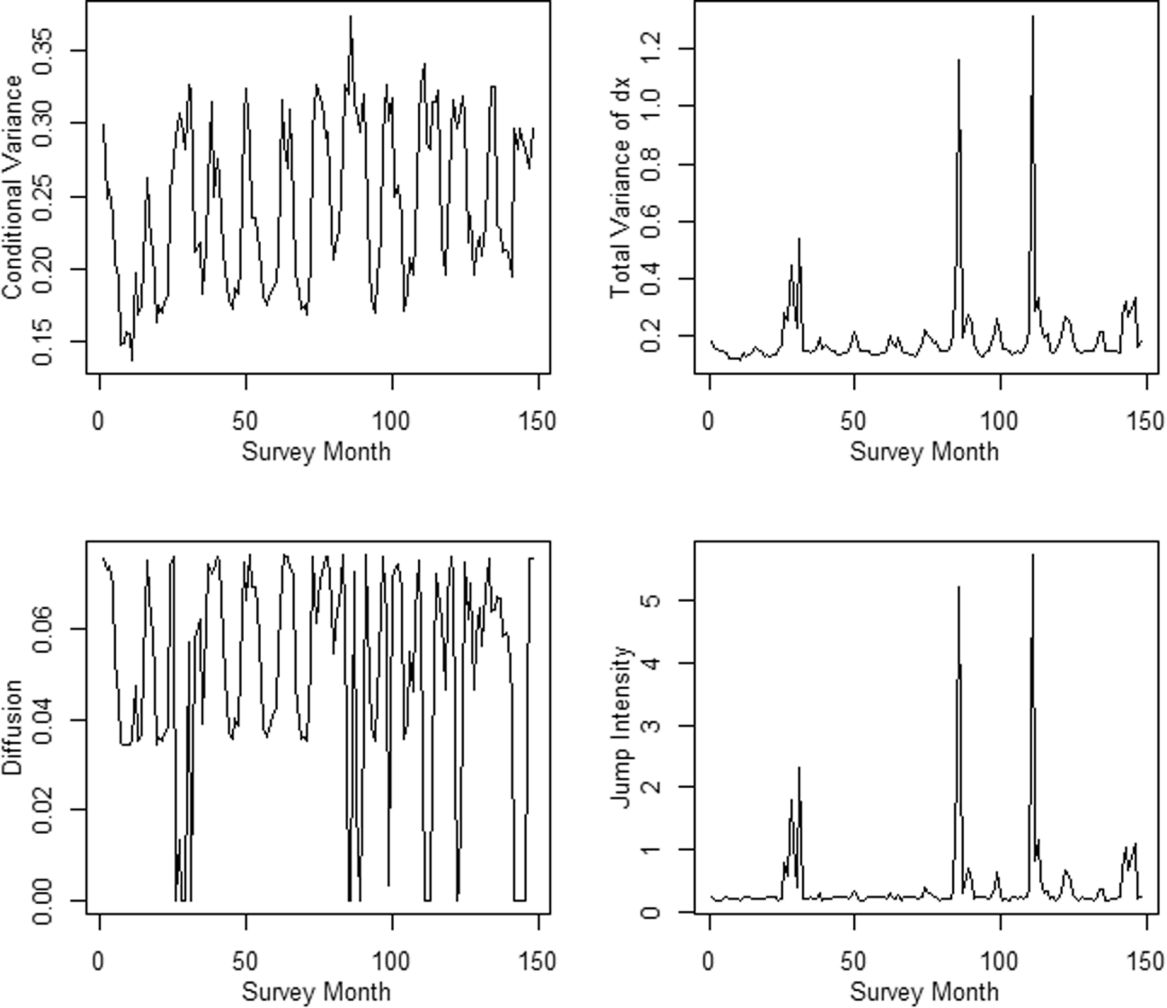
Results of the diffusion-drift-jump analysis of the spectacled flying-fox monitoring data. The distribution of (**a**) conditional variance, (**b**) total variance, (**c**) diffusion, and, (**d**) jump intensity as a function of time.

Drift-diffusion-jump modelling identified a slight increase in total variance in the middle of each year (Fig. 2). The size of these peaks increase moderately in 2014 and 2016 but three major peaks are clearly identified in mid-2006, 2011, and 2013. These peaks correspond with the cooler months immediately following the arrival of cyclones earlier in the year.

### Population modelling

#### Parameter estimates

Approximately 86% of spectacled flying-foxes are estimated to be found in camps during the month of December and only approximately 30% during the month of June.

The month-to-month estimate of log-normal process noise ($\widehat{{\sigma }_{proc}}=0.149$, 95% C.I. 0.99–0.21) is equivalent to a monthly percentage change in the order of 15%. This is considerable, and highlights just how noisy the processes underlying these data are. In fact it is similar to the model-based estimate of the mean counting precision of observers ($\widehat{{\sigma }_{obs}}\mathrm{=0.096}$, 95% C.I. 0.05–0.19).

**Figure 3:**
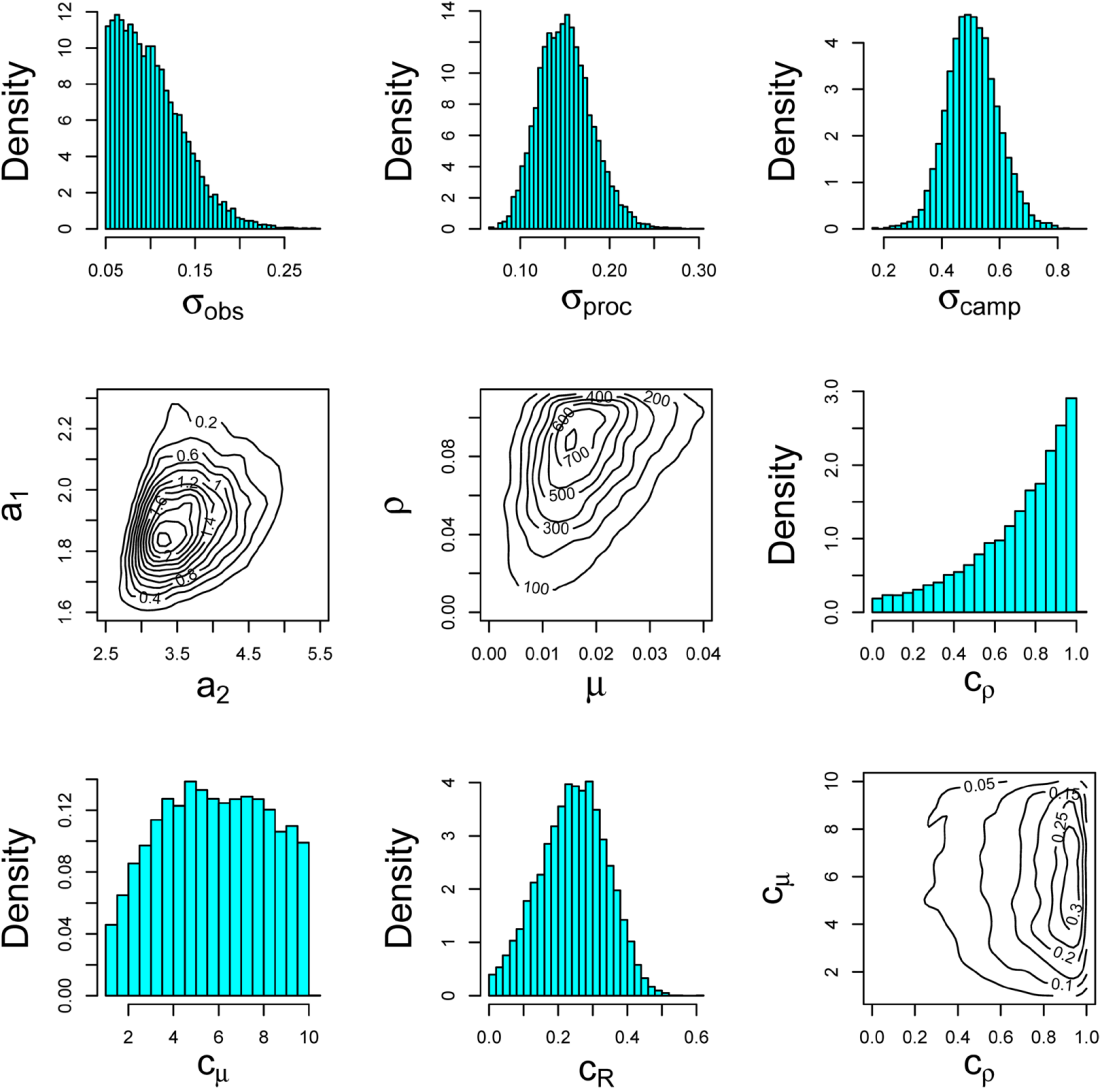
Posterior distributions for model parameters describing observation uncertainty (*σ*_*obs*_), process noise (*σ*_*proc*_), seasonality of roosting aggregation (*σ*_*camp*_), amplitude of seasonality (*α*_1_, *α*_2_), exponential rate of recruitment over three months (*ρ*) and mortality rate (*μ*) jointly, cyclone multiplicative effect on recruitment (*c*_*ρ*_), immediate cyclone multiplicative effect on mortality rate (*c*_*μ*_), cyclone multiplicative effect on the proportion in camps (*c*_*R*_), and relationship between *c*_*ρ*_ and *c*_*μ*_, expressed jointly.

The rate of recruitment and mortality are not easily individually identifiable from the data, and there is a ridge in the joint posterior density of the two (Fig. 3). This is not of concern, as we’re primarily interested in their joint effects on the population rate of increase (see below).

Cyclones were estimated to have a big impact on the population in a number of ways. The immediate effect was for the population to be dispersed away from camps, with the proportion of individuals roosting in camps immediately reduced by about a quarter (24%) from what would be there otherwise ($\widehat{{c}_{R}}=0.24$, 95% C.I. = 0.05–0.42) before recovering over the subsequent year. The initial (maximum) increase in the monthly mortality rate was estimated to be nearly 6-fold ($\widehat{{c}_{\mu }}\mathrm{=5.7}$, 95% C.I. = 1.5–9.7) the back ground rate ($\widehat{\mu }=0.017$ mth^−1^, 95% C.I. = 0.0044–0.038 mth^−1^). The effect of cyclones was still apparent in the subsequent breeding season, with the recruitment rate reduced by 21%, though the magnitude of the reduction was highly uncertain ($\widehat{{c}_{\rho }}\mathrm{=0.79}$, 95% C.I. 11.5–99). Posterior distributions of the model parameters are presented in Fig. 3. Populations recovered strongly after cyclones Larry and Yasi, but less so after cyclone Oswald (Fig. 1).

#### Rate of increase

There is a general visual trend for a decrease in the inferred numbers of flying foxes which is paralleled in the counted total population (Fig. 1). The estimated November population decreased from *c*. 326,000 (95% C.I. 244,000–493,000) in 2004 to *c*. 78,000 (95% C.I. c. 49,500–122,000) (Fig. 1) in 2017—a substantial decline.

**Figure 4:**
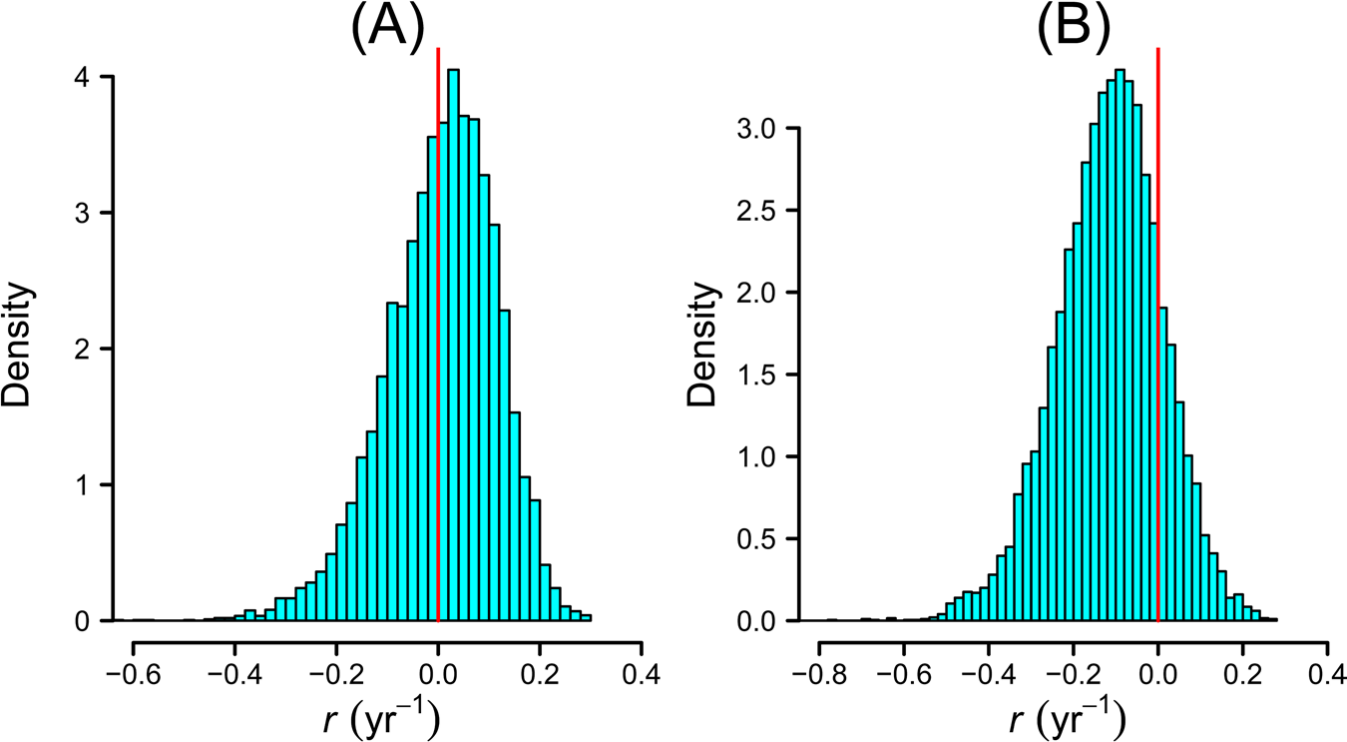
Posterior density distributions for the average yearly exponential rate of population increase (*r*). (**A**) In the absence of cyclones, and (**B**) in the presence of cyclones at the frequency observed during this study. Red vertical lines indicates no change in population (*r* = 0).

In the absence of cyclones, the population was estimated to have no discernible trend, with the estimated mean population rate of increase just above zero (Fig. 4A). Notably though, the most recent yearly decline from an estimated c. 154,000 individuals (95% C.I. c. 112,000–239,000) in November 2015 to an estimated c. 78,000 individuals (95% C.I. c. 50,000–122,000) is not associated with any cyclone (Fig. 1). In the presence of cyclones (averaged effect over study period), the population rate of increase was estimated to most likely be in decline (mean = −0.12 yr^−1^, 95% C.I. −0.39–0.11, Fig. 4B).

The posterior probability (our model-based belief) of a declining population over the period of the study (i.e. *P*(*r* < 0)) is 83%.

## Discussion

Monthly monitoring conducted over a period of 154 months (12.8 years) and at every known *P*. *conspicillatus* camp in the species’ Wet Tropics range showed two distinct trends. These were i) strong seasonal fluctuations in the population size, and, ii) a decline in total population over the period of monitoring (Fig. 1).

The marked seasonal fluctuations, with high numbers of animals counted in camps during the warmer months and lower numbers counted in the cooler months of each year, are perhaps the defining feature of the species’ population dynamics. Telemetry studies of *P*. *conspicillatus* reveal that animals are moving into and out of the counted population on a seasonal basis, with individuals spending 80% of days roosting in known camps during the warmer months compared with as little as 7% of days roosting in known camps during the cooler months^21^. Other possible explanations, that the dynamics reflect mortality and recruitment processes or movement in and out of the species’ range, are not supported. There was no evidence of mass mortality or the capacity to recover from such events within a year, and, just one tagged animal was recorded foraging outside the normal range and then only briefly. Taken together these observations make it seem likely that the observed seasonal fluctuations result from movement to small and ephemeral roosts within the species’ normal range during the cooler months.

The strong seasonal fluctuations in estimated abundance that result from these changes in aggregation add to the difficulty of confidently detecting trends. Traditional analytical approaches are not well suited to this situation. The predominant historical trend estimation method, log-linear regression of abundance data against time^28,29^, ignores the temporal dependence of the data, as well as the possibility of process noise that goes with this^30^. The outcome is that precision is invariably overestimated, along with the statistical power to detect trends in population size. The use of state-space modelling allows both observation and process noise to be modelled within a single process and framework. This has the advantage of effectively increasing the confidence in the inferences made from it while the Bayesian estimation approach enables a more straightforward interpretation of the results.

Overall, the state-space model corroborates the analysis of the raw data in showing a total population that declines in a series of steep steps followed by partial recovery (Fig. 1). Furthermore, the fit of the model to the observed data is good and its prediction, that the population is likely to remain at low levels, should be of concern for management.

The early warning analysis^31,32^ proved an effective tool for identifying critical transitions in the variance structure of this temporally correlated data set, and, by identifying the timing of these transitions, provided insights into the underlying drivers of the decline. This was critical for this study where not only was the decline itself initially difficult to detect but where the underlying drivers were also obscured by the annual fluctuations and noise in the population time-series.

The drift-diffusion-jump modelling identified perturbations, occuring during the cooler months of the year, at two scales. In nine years these perturbations were minor and most likely simply reflected the annual movement of animals out of the counted population or possibly the loss of animals during the lean, cooler months (Fig. 2). In 2014 and 2016 these perturbations were moderate, suggesting that perhaps movement out of camps was greater during these periods or that resources were scarcer. No significant events can be ascribed to these years, thus their cause is unclear.

Major perturbations were recorded mid-year in 2006, 2011, and 2013 in the cooler months following a major cyclone earlier in the year. These cyclones were Cyclones Larry (Category 4, 20 March 2006), Yasi (Category 5, 3 February 2011) and Oswald (Category 1, 23 to 24th January 2013). Both Larry and Yasi were severe cyclones that caused extensive and major damage to forests across a large swathe of the central and southern sections of *P*. *conspicillatus*’ range. With wind speeds of up to 240 and 250 km h^−1^, respectively, these cyclones snapped trunks, uprooted trees, and stripped major branches and most foliage up to 30 km from the central track and caused foliage damage extending well beyond this^33,34^. Some *P*. *conspicillatus* in the path of these cyclones would have inevitably been killed as a direct result of these winds but the extensive damage to the forest canopy would also have had significant and long-term impacts on fruit and flowering resource availability^35^, and likely resulted in significant indirect mortality over subsequent months.

Cyclone Oswald was more modest with wind speeds reaching only 140 km h^−1^ and the damage to forests being largely confined to the stripping of leaves, fruits and flowers, and the loss of minor branches. However, Oswald travelled from north to south on the coast and, as a consequence, its impact was exerted across almost the entire Wet Tropics range of *P*. *conspicillatus*. While it is unlikely that there was significant direct *P*. *conspicillatus* mortality associated with Cyclone Oswald, it is likely that its impacts on foraging resources over the subsequent 12 months resulted in significant indirect mortality^35^.

The early warning analysis suggests that tropical cyclones play a key role in modulating *P*. *conspicillatus* population dynamics with the lag in the onset of the perturbations (Fig. 2) pointing to their influence being exerted primarily through indirect effects, i.e. the loss of fruit and flowering resources during the cooler months of the year. While tropical cyclones have been identified as drivers of population dynamics in insular flying-fox populations in the past^36,37^ this is the first study to document such effects in a continental context. With intense cyclones predicted to increase in frequency and severity under climate change^38,39^, they take their place along with other extreme climate events^24^ as conservation threats to mainland flying-fox populations. The impact of this succession of cyclones on *P*. *conspicillatus*’ abundance suggests that similar but largely un-noted effects may have occurred for other frugivore and nectarivore species and raises questions about the resilience of these guilds under future climate change.

Should there be concern about this decline or is *P*. *conspicillatus’* population simply declining from an unusually high level after a period free from cyclones? Three points suggest that there is reason for concern. First, between 1878 and 2006 eleven “significant” cyclones impacted the Wet Tropics region with an average return time of *c*. 12 years^33^. Significant cyclones made landfall in the region five and 18 years prior to this study suggesting that the population was not experiencing a period of unusual respite. Second, the population levels recorded at the beginning of this study were similar to those estimated in the only previous census of the population. This was a partial census conducted in 1998 and counted a population of 150,000 individuals (± 30,000) for a total estimated population of 200,000^40^. Third, two of the three most intense cyclones recorded in the region occured during a five year period during this study. Combined this suggests that the current decline is unusual and not a population being reduced to normal levels.

Our results have considerable implications for the species’ status. Under Australia’s Environment Protection and Biodiversity Conservation Act (EPBC) (1999) a decline of >50% and < 70% over the longest of either 3 generations or 10 years is required to warrant listing as Endangered. This decline can be observed, inferred or projected. Here we are describing a 75% decline over a 13.8 year period. Current estimates of the duration of three *P*. *conspicillatus* generations range from 15 years^41^ to 24 years^42^ depending on the method used. Irrespective of which generation time we use *P*. *conspicillatus* qualifies for listing as Endangered, and arguably for listing as Critically Endangered, under the EPBC Act.

It must be noted however that while the decline is currently occurring, whether it continues is unclear. Cyclones are not new in the Wet Tropics Region and *P*. *conspicillatus* has presumably recovered from their impacts in the past. If, as our data suggest, cyclones are the primary driver of the decline, then whether it continues will depend on: i) when the next significant cyclone hits the region, and, ii) what impact other threats have on the species over the coming years. The continued decline in the absence of cyclones in the last years of the monitoring points to the possibility that the population may have reached a level where other threats are now keeping it low. These may include one or more of the following; an increase in clearing that occurred in the region during the period 2013–2015^43^, an increasing frequency of extreme temperature events^24^, paralysis tick attack^41,44^, persecution in orchards^45^, and the disruption of urban camps^15^. There is currently little evidence to directly link any of these threats to the recent declines, however, unlike cyclones, each of these threats can be managed and should therefore be a focus of management efforts.

As anthropogenic pressures on ecosystems and species grow, managers will increasingly need to infer trends in the abundance of species, such as flying-foxes, from incomplete and noisy monitoring data. This study confirms a role for state-space modelling in providing estimates of total abundance that are statistically conditioned on available monitoring data and knowledge of population processes. We have developed a concise modelling framework that accounts for multiple potential sources of uncertainty, time varying populations available for monitoring, and severe weather events. Central to this is an integrated approach to the accounting of monitoring biases through the use of a process model to account for the dynamic nature of flying-fox populations and its associated uncertainty, and a data model to account for the uncertainty in the monitoring counts.

Given the population decline we have described here, the uncertainty about threats into the future, the loss of foraging habitat in drier parts of the range^43^ and the unfortunate timing of increased urbanisation in the *P*. *conspicillatus* population^15^, we suggest that *P*. *conspicillatus* should be listed as Endangered under Australia’s EPBC Act.

## Methods

### Data collection and background

Since May of 2004, all spectacled flying-foxes in known camps within the Wet Tropics region of Queensland, Australia, have been counted monthly. All known camps in the region (N = 64) are censused over a three-day period with an average of 10 (±3.08 S.D.) occupied in a given month. The choice of counting method used at a camp is determined by the physical access to the camp and whether the animals will tolerate the counter’s presence. The errors of these different methods are similar (e.g., their mean precision is 34%, ± 5)^21^ and the methods are outlined in detail in^22^ and^21^. When the camp is small (<1000 individuals) and provides good visibility, direct counts of all animals are conducted. Where access to the boundary is not possible, e.g., the camp is in mangroves, then a fly-out count is conducted. Where camps are large (generally >1000) and access or a good view of the camp is possible some form of sub-sampling is conducted; distance sampling^7^ when access through the camp interior is possible and tolerated, or a density estimate based on sample of trees or a defined area if not. This density estimate is then multiplied by the area of the camp or number of trees to get an estimate of the size of the camp. Camp area is determined by walking the camp boundary and recording its location and error using the Android application GeoMeasure by ObjectGraph.

This monitoring was conducted under Scientific Purposes Permits and Section 173p Permits from the Queensland Department of Environment and Heritage Protection and Animal Ethics Approvals from the CSIRO Wildlife and Large Animal Animal Ethics Committee. All aspects of the work were conducted in accordance with the relevant guidelines and regulations of the jurisdictions in which it was conducted.

### Early Warning Analysis

To identify whether the population time series data generated by the monitoring program exhibited any critical transitions, and if so, to identify the timing of these transitions, we used nonparametric drift-diffusion-jump modelling^31^. Drift-diffusion-jump modelling identifies changes in the structure of time-series data by fitting general models that approximate a wide range of non-linear processes at different points in the data and documenting the effect on the fit^32^. Here, we use drift-diffusion-jump modelling to identify the timing of significant perturbations in the *P*. *conspicillatus* population data. Analyses were conducted using the “earlywarnings” package in R^31^.

### Model description

SSMs are increasingly being used in ecology for population dynamics and estimation^7,46,47^. We propose a SSM for flying-fox population estimation that incorporates observation error, seasonal recruitment to the potentially countable (modelled as latent) population, a time-varying proportion of the total population available for counting (i.e. in a known camp), and major weather disturbances (impacting on the proportion of individuals in known camps, mortality, and recruitment). We use a discrete time population growth model. We do not choose a logistic-type model (e.g. Ricker), as this assumes a carrying capacity exists for the population, with associated density-dependent population growth rate. The total abundance for the spectacled flying-fox data is considered a latent variable. Process noise is incorporated into the model using a log-normal distribution and process noise variance, ${\sigma }_{proc}^{2}$. At any given time *t*, the total population of spectacled flying-foxes, *X*_*t*_, is modelled as

1 $${X}_{t} \sim \,\mathrm{ln}\,{\mathscr{N}}(\mathrm{log}({X}_{t-1}\exp ({\rho }_{t}-{\mu }_{t})),{\sigma }_{proc}^{2}),$$

where, $\mathrm{ln}\,{\mathscr{N}}\,({\theta }_{1},{\theta }_{2})$ is the log-normal distribution with mean *θ*_1_ and variance *θ*_2_, *ρ*_*t*_ is the recruitment to the countable population (over December-February), and *μ*_*t*_ is the mortality rate. Here, *t* is measured in months.

In any given month, some proportion of spectacled flying-foxes will be unavailable to be counted in camps. This could be due to the animals roosting in unknown camps or the animals not sleeping in a camp. The second of these potential absences from camps is seasonal in nature as more spectacled flying-foxes are found in camps during the mating (summer) season. We use a simple cosine function to model this seasonal behaviour.

Three major cyclones occurred during the data observation period; Cyclone Larry in March 2006, Cyclone Yasi in February 2011 and Cyclone Oswald in February 2013. The results of the early warning analysis (see Results below) clearly indicated cyclones as causing disturbance to the population counts. To account for cyclone effects we introduce an additional parameter, *c*_*R*_∈ (0, 1), which is the additional proportion of individuals not choosing to roost in large camps as a result of cyclonic disturbance. We also incorporate an in-camps process noise ${\sigma }_{camp}^{2}$ on a logit scale, which accounts for the inevitable reality that the population won’t be tracking the modelled seasonal trend exactly from year-to-year (i.e. flying fox behaviour is not deterministic). The proportion of spectacled flying-foxes in known camps at time *t*, ${X}_{t}^{C}$, is thus modelled as

2 $${X}_{t}^{C} \sim \,\mathrm{ln}\,{\mathscr{N}}\,(\mathrm{log}({p}_{t}{X}_{t}),{\sigma }_{camp}^{2}),{\rm{with}}$$

3 $${p}_{t}=\frac{\cos (\frac{2\pi t}{12})+{\alpha }_{1}}{{\alpha }_{2}}\mathrm{(1}-{c}_{R}{I}_{C}),$$

where, *I*_*C*_ is an indicator function that is unity (one) for the year of severe tropical cyclones, and zero otherwise. Here, *α*_1_ ∈ (1, inf) and *α*_2_ ∈ (*α*_1_ + 1, inf) are parameters that govern the seasonal proportion of spectacled flying-foxes in camps. In non-cyclone years, the proportion *p*_*t*_ is largest in December, $\frac{{\alpha }_{1}+1}{{\alpha }_{2}}$, and lowest in June, $\frac{{\alpha }_{1}-1}{{\alpha }_{2}}$. We also modelled the effect of cyclones on the subsequent recruitment season using a multiplicative parameter *c*_*ρ*_, bounded between 0 (nil recruitment) and 1 (no effect on recruitment). Additional cyclone-induced mortality was also modelled as a multiplicative factor *c*_*μ*_, with a lower bound of 1 (no effect). The cyclone effect was modelled as an immediate step down following by a linear ramp back to normal conditions after 12 months.

Observed counts of spectacled flying-foxes in camp *i *∈* I* at time *t*, *X*_*t*,*i*_, where *I* is the set of all known camps, are summed to get the observed count at time *t*, *Y*_*t*_ = ∑_*i*∈*I*_ *X*_*t*,*i*_. The observation count *Y*_*t*_ is assumed to be log-normally distributed:

4 $${Y}_{t} \sim \,\mathrm{ln}\,{\mathscr{N}}(\mathrm{log}({X}_{t}^{C}),{\sigma }_{obs}^{2})\mathrm{.}$$

Here, ${\sigma }_{obs}^{2}$ is the observation error.

### Parameter estimation

A Bayesian hierarchical modelling approach was used to estimate the unknown state variables *X*_*t*_ and ${X}_{t}^{C}$, as well as the unknown parameters *ρ*, *c*_*ρ*_, *μ*, *c*_*μ*_ *α*_1_, *α*_2_, ${\sigma }_{obs}^{2}$, ${\sigma }_{t}^{2}$, ${\sigma }_{C}^{2}$ and *c*_*R*_. The model fitting was undertaken within the R software environment^48^ using the rjags package^49^.

Priors used for the parameters are found in Table 1. Where possible, we incorporated prior information (beliefs) with the distributional form and parameterisation either informed from previous studies (e.g. for maximum rate of recruitment consistent with maximum rate of yearly population increase) or model assumptions (e.g. cyclones can only increase mortality). Otherwise prior distributions were chosen as “flat”, which we recognize is not necessarily equivalent to being uninformative. Three chains of 50,000 steps were run following an initial “burn-in” period of 10,000 steps. Convergence of chains was assessed using the convergence diagnostic of Gelman and Rubin^50^ that calculates potential scale reduction factors. Potential scale reduction factors were very close to 1 (indicating convergence) for all parameters other than the in-camp process uncertainty (*σ*_*camp*_) that was 1.06, but still well within the value of 1.2, which is sometimes used to indicate “approximate convergence”^51^. We are comfortable with this, as our primary interest is the population rate of increase, and not predicting the seasonal dynamics we are comfortable with this outcome.

**Table 1:** Prior distributions of the model parameters.

Parameter	Symbol	Prior	Rationale
Rate of recruitment (mth^−1^)	*ρ*	*U* (0, 0.11)	Maximum 40% increase (over 3 months)
Mortality rate (mth^−1^)	*μ*	*U* (0, 0.1)	Maximum 70% reduction (over 12 months)
Cyclone effect on mortality	*c*_*μ*_	*U* (1, 10)	Initial 10-fold increase the maximum believed possible
Cyclone effect on recruitment	*c*_*ρ*_	*B* (1.01, 1.01)	Largely uninformative
Observation uncertainty std dev. (lognormal)	${\sigma }_{obs}^{2}$	*U* (0.05, 0.47)	Equates to belief CV of between 5% and 50%^27^
Process uncertainty std dev. (lognormal)	*σ*_*proc*_	*U* (0, 10)	Uninformative (on logit scale)
Seasonality parameter 1	*α*_1_	*U* (1, ∞)	Uninformative
Seasonality parameter 2	*α*_2_	*U* (*α*_1_ + 1, ∞)	Uninformative
Cyclone in-camp roosting effect	*c*_*R*_	*B* (1.01, 1.01)	Uninformative, other than down-weighting 0 (total abandonment) and 1 (no effect)
In-camp process uncertainty std dev. (normal on logit scale)	*σ*_*camp*_	*U* (0, 100)	Uninformative
Exponential rate of increase (yr^−1^)	*r*	—	Calculated from the posterior distribution based on *ρ*, *c*_*ρ*_, *μ*, *c*_*μ*_ and observed cyclone frequency

Here, *U*(*θ*_1_, *θ*_2_) is the uniform distribution with endpoints *θ*_1_ and *θ*_2_ and *B*(*θ*_1_, *θ*_2_) is the beta distribution with shape parameter *θ*_1_ and scale parameter *θ*_2_.

We make the assumption that all spectacled flying-fox camps in the Wet Tropics are known. Our long-term monitoring program, combined with a concurrent GPS telemetry study of 63 individuals and public reporting make this a reasonable assumption^21^. We analyzed the abundance estimates of the final available time point (February 2017) as this provides the current estimate of spectacled flying-fox abundance.

The datasets generated during and/or analysed during the current study are available from the corresponding author on reasonable request.
